# Towards Dynamic Remote Data Auditing in Computational Clouds

**DOI:** 10.1155/2014/269357

**Published:** 2014-07-09

**Authors:** Mehdi Sookhak, Adnan Akhunzada, Abdullah Gani, Muhammad Khurram Khan, Nor Badrul Anuar

**Affiliations:** ^1^Center for Mobile Cloud Computing Research (C4MCCR), University of Malaya, 50603 Kuala Lumpur, Malaysia; ^2^Center of Excellence in Information Assurance (CoEIA), King Saud University, Riyadh 92144, Saudi Arabia; ^3^Department of Computer System and Technology, Faculty of Computer Science and Information Technology, University of Malaya, 50603 Kuala Lumpur, Malaysia

## Abstract

Cloud computing is a significant shift of computational paradigm where computing as a utility and storing data remotely have a great potential. Enterprise and businesses are now more interested in outsourcing their data to the cloud to lessen the burden of local data storage and maintenance. However, the outsourced data and the computation outcomes are not continuously trustworthy due to the lack of control and physical possession of the data owners. To better streamline this issue, researchers have now focused on designing remote data auditing (RDA) techniques. The majority of these techniques, however, are only applicable for static archive data and are not subject to audit the dynamically updated outsourced data. We propose an effectual RDA technique based on algebraic signature properties for cloud storage system and also present a new data structure capable of efficiently supporting dynamic data operations like append, insert, modify, and delete. Moreover, this data structure empowers our method to be applicable for large-scale data with minimum computation cost. The comparative analysis with the state-of-the-art RDA schemes shows that the proposed scheme is secure and highly efficient in terms of the computation and communication overhead on the auditor and server.

## 1. Introduction

Despite being a promising business concept, cloud computing is also becoming the fastest growing segment of the IT industry. It is all about moving services, computation, and/or data off-site to an internal or external, location-transparent facility or contractor. It is the way to increase the capacity or to add capabilities without investing in new infrastructure, licensing new software, or training new personnel. Despite many existent cloud definitions, they all agree that this paradigm aims at offering every network-accessible computing resource “as-a-service” (XaaS); however, the most highly structured definition comes from the National Institute of Standards and Technology (NIST) [1–3]. Thus, cloud computing is a key technology for empowering convenient, on-demand network access to a shared pool of configurable computing resources with negligible service provider interaction or management effort. Therefore, enterprise and businesses tend to outsource their data on the cloud storage without investing in extra hardware, software, and the maintenance [4].

Despite the fact that cloud offers noticeable services for data owners, storing data to a remote server and entrusting management of data to a third party result in losing the physical control over the data [5, 6]. Though cloud has a promising, resilient, and reliable architecture, the data in the cloud is still susceptible to many threats and encounters many security challenges. It might lead to compromise the confidentiality, integrity, and availability of data. Examples are included to be able to delete less frequently accessed data to make available disk space or to hide data damage in order to protect the reputation Recently, owners may lose their outsourced data on the cloud due to service and data disruptions in servers with major cloud infrastructure providers such as Amazon S3 breakdown [7], Gmail email mass deletion [8], Sidekick Cloud Disaster [9], and Amazon EC2 service's outage [10, 11]. Besides, more than 535 data breaches in 2011 were reported with breaching of a cloud-based email service provider in Epsilon [12], stealing 3.3 million patients' medical details of Sutter Physicians Services, a major compromise of Sony PlayStation Network, Sony Online Entertainment, and Sony Pictures, stealing customers' information on EMC's RSA, exposing 150 million user accounts of Adobe Company, and most importantly leaking more than 104,000 employees' and contractors' private information of the Department of Energy in the U.S. that are the good examples of the data breach in 2013 [13]. Subsequently, the owners may have a high tendency to lose their outsourced data on the cloud.

To address this important issue, several researchers have proposed remote data auditing (RDA) protocols (e.g., [14–16]) to securely, frequently, and efficiently verify the integrity of the data stored over a cloud. RDA schemes mainly fall into three main categories: (1) integrity-based: it actually enables a cloud user to verify the integrity of data, (2) recovery based: data recovery is performed by leveraging error correction and erasure codes; however, normal integrity verification provides a way for recovering data in case of any possible corruption, and (3) deduplication-based: it is meant to improve the efficiency of data storage and mitigate the communication overhead of data outsourcing.

However, designing a proper remote data auditing mechanism, a set of noteworthy properties must be taken into consideration. These properties are as follows. (1) Efficiency: it is to verify data with a minimum possible amount of computational time, storage space, and communication between client and server. (2) Mode of verifiability (public/private): private verification methods exclusively work on the client's computer; however, in a public verification, the intricate task of verification is delegated to a third party often called third party auditor (TPA). The rationale behind this delegation is to take advantage of expertise and large capabilities of TPA as compared to limited computing power of client machine. (3) Frequency: it is the maximum number of times a user can verify his data. (4) Probability of detection: it represents the probability by which a potential data loss is discovered. (5) Dynamic update: it is the ability to verify the integrity of data without downloading the whole data when data is liable to different kinds of update operations, including insert, delete, modify, and append.

This paper proposes an efficient remote data auditing method based on an algebraic signature which allows the client to check data possession in cloud storage efficiently while incurring less computation overhead on cloud side and client side compared to homomorphic cryptosystem. Furthermore, we extend our data auditing scheme by designing an efficient data structure to support dynamic data update feature with minimum computation overhead on client and cloud server. The contribution of this paper is summarized as follows.

1. We propose an efficient remote data auditing scheme for data storage in cloud computing based on algebraic signature. Our data auditing scheme incurs the minimum computation and communication cost on client and cloud server.
2. We design a new data structure to efficiently support dynamic data operations, such as insert, append, delete, and modify operations. This data structure empowers our method to be applicable for large scale data with least computation cost on client and server.
3. We implement our scheme to prove the security, justify the performance of our method, and compare with the stat of the art data auditing methods.

The rest of the paper is organized as follows.  Section 2 discusses the related work.  Section 3 introduces the preliminaries and the fundamental concepts which are used in the construction of our method. In Section 4, we introduce the details of our remote data auditing scheme. We describe the security analysis of our scheme in Sections 5 and 6 gives the performance analysis in terms of computation overhead. Finally, the conclusion of this paper is presented in Section 7.

## 2. Related Work

Recently, a great deal of attention has been paid to the RDA schemes that are used to check the correctness of outsourced data in cloud computing [15–22].

Ateniese et al. [15] were the first to propose the provable data possession (PDP) scheme to check the correctness of the outsourced data statically in the cloud storage without having to retrieve the data. They used the RSA-based homomorphic verifiable tag to combine the tags and to build a proof message that permits the client to check whether the server has specific blocks, even when the client has no access to the blocks. However, the PDP scheme incurs high computation and communication cost on the server side due to the usage of RSA numbering over the whole file. It also has linear storage for the user and fails to provide secure data possession when the server has a malicious intent [23, 24]. In [16], Ateniese et al. considered static data update issue in the original PDP method [15] and developed a semidynamic data auditing method based on symmetric-key operations. This method allows a user to modify, delete, or append the stored data in the cloud. However, the data owner needs to regenerate all remaining challenges during the update operation, which makes it inapplicable for huge files.

Jules and Kaliski [25] defined a type of the RDA techniques, namely, proof Of retrievability (POR) in which an auditor has also the capability to recover and mitigate data corruption by using forward error-correcting codes when data is stored in untrusted cloud. To achieve this goal, the data owner needs to create a set of sentinel blocks by using a one-way function and inserts the sentinels randomly on the data blocks before uploading to the server. If the server modifies a small portion of the file, the verifier can find it and check the integrity of a file easily due to the effect of file modification on the sentinels. However, the number of queries in such method depends on the number of inserted sentinel blocks. Moreover, the POR method incurs high computation overhead on the client side because of the error recovery and data encryption processes. The work proposed by Shacham and Waters [26] improved the efficiency and security of the original POR based on the data fragmentation concept. The authors used the BLS homomorphic authentication [27] to generate a fixed size tag by aggregating all of the tags to minimize the network computation cost and used the Reed Solomon code to recover the corrupted blocks. The main disadvantage of this method is supporting static data update. Furthermore, during public verification process, the privacy of data cannot be protected against a trusted third party. The majority of POR methods failed to efficiently support dynamic data update because the server is unable to realize the relation between the data blocks and encrypted code words. Cash et al. [28] addressed this issue and designed a first dynamic POR scheme by using the ORAM technique [29]. The dynamic POR method also incurs high computation overhead on the client and server side.

The work by Erway et al. [18] addressed the dynamicity issue in the PDP schemes by combining the skip list [30], rank-based information, and authentication dictionary. Each node in this data structure needs to store the number of reachable nodes from this node as a rank. Although the dynamic PDP method ensures the integrity of variable-sized data blocks, it is unable to verify the integrity of individual block [31].

Wang et al. [19] employed a combination of the Merkle hash tree (MHT) [32] and bilinear aggregate signature [27] to propose a dynamic remote data auditing in cloud computing. The main contribution of this method is in manipulating the classic MHT construction by sorting the leaf nodes from left to right in order to support dynamic update and determine the insert, delete, or modify positions by following this sequence and computing the root in MHT. However, the method leaks the data content to the auditor and incurs heavy computation cost on the auditor.

Yang and Jia [17] implemented an efficient data auditing scheme to overcome the privacy issue in [19]. The authors used the bilinearity property of the bilinear pairing for generating an encrypted proof such that the auditor is only able to verify it. They also design a new data structure to support dynamic operations in which data owner needs to store a row, including block index and block logical location for each block of outsource file. During the delete and insert operations, the auditor has to find the position of the required block (*i*) and shift the remaining blocks (*n* − *i*) to create or delete a row in such data structure. However, by increasing the number of blocks in the data structure, the auditor needs to shift a huge number of blocks, which incur the high computation overhead on the auditor. The other drawback of this method is that deleting or inserting a large data block imposes high computational overhead on the auditor side. Furthermore, the bilinear pairing computation is more expensive than the algebraic structure that is used in our method [33, 34].

## 3. Preliminaries

This section provides an overview on the background of our dynamic remote data auditing method. We first describe the general architecture of the remote data auditing protocol. Then, we state the fundamental technique of this method that is called algebraic signature in order to audit the outsourced data efficiently.

### 3.1. System Model

**Figure 1:**
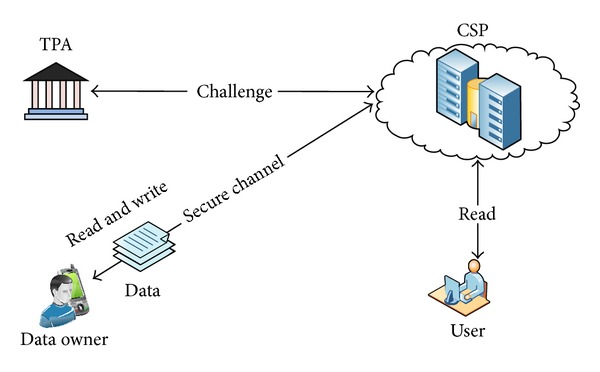
The network architecture of RDA in cloud computing.

The architecture of RDA protocols in a network comprises four key entities: (1) user: it represents an enterprise or individual having permission to read the stored data in the cloud, (2) data owner (DO):it is enterprise or businesses which store their data in the cloud storage having the ability to do update operations (modify, delete, and insert), (3) cloud storage provider (CSP): this entity is responsible to back up the user data and generates a proof as a response of the received challenges, and (4) third party auditor (TPA): auditing the outsourced data and its verification is done by TPA. It actually ensures whether the data remains intact over the passage of time in public auditing models. Private auditing schemes, however, cannot support the TPA and DOs in order to check the integrity of the data.  Figure 1 clearly depicts the typical RDA components and their interactions.

### 3.2. Algebraic Signatures

Algebraic signature is a type of hash functions with algebraic properties that allows computing the signatures of unseen messages in a limited way. The fundamental feature of algebraic signature schemes is to take a signature of the sum of some random blocks giving the same result as taking the sum of the signatures of the corresponding blocks [35].

Let an element *γ* in the Galois field be composed of a vector of various nonzero elements *γ* = (*γ*_0_, *γ*_2_,…, *γ*_*n*−1_). An algebraic signature of file *F* including *n* data block (*f*[1], *f*[2],…, [*f*[*n*]) is computed by

(1) $$\begin{array}{c} S_{\gamma }\left(F\right)=\overset{n}{\underset{i=1}{\sum }}f\left[i\right]\cdot \gamma^{i-1}. \end{array}$$

In the following, a number of algebraic signature properties are listed.

**Proposition 1 .**

Litwin and Schwarz [36] have also shown that the algebraic signature of concatenation of two blocks *b*[1] with length *r* and *b*[2] is computed by

(2) $$\begin{array}{c} S_{\gamma }\left(f\left[i\right]||f\left[j\right]\right)=S_{\gamma }\left(f\left[i\right]\right)⊕r^{\gamma }S_{\gamma }\left(f\left[j\right]\right). \end{array}$$

**Proposition 2 .**

The algebraic signature of summation of blocks of a file *F* is equal to summation of signature of each of the blocks

(3) Sγ(f[1])+Sγ(f[2])+⋯+Sγ(f[n]) =Sγ(f[1]+f[2]+⋯+f[n]).

**Proof**

Assume that the file *F* is divided into *n* blocks and each of the block consists of *r* sectors. Then,

(4) Sγ(f[1])+Sγ(f[2])+⋯+Sγ(f[n]) =∑j=1rf[1][j]·γj−1+∑j=1rf[2][j]·γj−1  +⋯+∑j=1rf[n][j]·γj−1 =∑j=1rγj−1(f[1][j]+f[2][j]+⋯+f[n][j]) =Sγ(f[1]+f[2]+⋯+f[n]),

where *f*[*i*][*j*] indicates the *j*th bit of block *i* in file *F*.

**Proposition 3 .**

The algebraic signature of summation of two files *F*,  *G* is equal to summation of signature of the files

(5) $$\begin{array}{c} S_{\gamma }\left(F+G\right)=S_{\gamma }\left(F\right)+S_{\gamma }\left(G\right). \end{array}$$

**Proof**

Assume that the files *F* and *G* include *n* blocks. Then, the summation of signature of such files can be computed by

(6) Sγ(F)+Sγ(G)=∑i=1nf[i]·γi−1+∑i=1ng[i]·γi−1=∑i=1nγi−1(f[i]+g[i])=Sγ(F+G).

## 4. The Proposed Scheme

This section presents the applied techniques and algorithms of our dynamic remote data auditing scheme. We also show the correctness proof of our method by using the characteristics of the algebraic signature technique.

### 4.1. Data Auditing Algorithm

Suppose that file *F* includes *n* data blocks and each of the block is divided into *r* sectors by using the data fragment technique. If the last block has less number of sectors, we increase the size of the block by setting *f*_*l*,*j*_ = 0 for *j* ≤ *r*. Our data storage auditing scheme consists of the following phases.

*Setup.* The DO firstly generates the public and secret key by executing the keygen algorithm (KeyGen(1^*k*^) → (*pk*, *sk*)). Then, the unique tag (metadata) for each block of input file is computed based on the algebraic signature of the block using the following formula:

(7) $$\begin{array}{c} T_{i}=S_{\gamma }\left(f\left[i\right]||\left({\text{ID}}_{F}||i||L_{i}||V_{i}^{}\right)\right), \end{array}$$

where *f*[*i*] is *i*th block of file *F*, ID_*F*_ is unique identity of the file, *L*_*i*_ is the logical number of file in the DCT table, and *V*_*i*_ indicates the version of data block. Also, the DO computes *C*_*i*_ = *S*_*γ*_(ID_*F*_||*i*||*L*_*i*_||*V*_*i*_) for each data block to prevent the replay attack. When all of the tags are generated, the DO outsources the data blocks along with the considering tags to the cloud {*f*[*i*], *T*_*i*_, C_*i*_}_*i*=1_^*n*^. 

*Challenge*. When the DO decides to check the correctness of the outsourced data, it selects *c* data blocks randomly as a challenge message (chal = {*cs*_*i*_}_*i*=1_^*c*^) by using pseudorandom permutation [37] keyed with a fresh randomly chosen key in order to prevent the server from anticipating the block indices.

**Proof**

Upon receiving the challenge message, the cloud computes a linear combination of the blocks (*σ*) and the aggregate authenticator tags (*μ*) as a proof message based on the received challenge and the corresponding tags by using

(8) $$\begin{array}{c} \mu =\overset{{cs}_{c}}{\underset{i={cs}_{1}}{\sum }}T_{i}⊕C_{i}\;\;\sigma =\overset{{cs}_{c}}{\underset{i={cs}_{1}}{\sum }}f\left[i\right]. \end{array}$$

*Verification*. Upon receiving the pair (*μ*, *σ*), the DO uses the algebraic signature of the block tags to verify the correctness of the blocks by using the following formula:

(9) $$\begin{array}{c} S_{\gamma }\left(\sigma \right)\overset{?}{=}\mu . \end{array}$$

### 4.2. Dynamic Data Operations

To support dynamic data update, we propose a data structure that is called Divide and Conquer Table (DCT). The DCT prevents the server from using the previous version of the stored data instead of the updated one to pass the verification phase (replay attack). The DCT consists of two components: logical index (*L*_*i*_) and version number (*V*_*i*_). The *L*_*i*_ indicates the original index of data block and the *V*_*i*_ indicates the current version of block on the basis of number of updates. When a data block is updated, the considered *V*_*i*_ in DCT must be incremented by 1. The index of each block in DCT also denotes the physical position of the outsourced data block.

This data structure must be created by the DO before outsourcing a data block to the cloud. The DO is in charge of managing the DCT during update operation. Therefore, by increasing the size of file, a huge computation overhead is imposed on the owner side. For example, to insert a new data block after the *i*th block, the data owner must shift *n* − *i* blocks, which waste the time and impose additional computation overhead. To overcome this issue, we reduce the size of the DCT by dividing it into *k* data structures in which each of them is able to store ⌈*n*/*k*⌉ of the data blocks. As a result, when the DO decides to insert a new block after the *i*th block, the data owner only needs to shift the ⌈*n*/*k*⌉ − *i* data block. The experimental results show that the proposed data structure is able to support the large scale data efficiently. In the rest of this section, we discuss how our scheme performs dynamic data operations, such as modify, insert, delete, and append.

*Data Modification*. One of the important requirements of remote data auditing techniques is to support the data modification operation in which the DO has capability to replace the specified blocks with new ones. Suppose that the DO wants to modify the *i*th block of the file *F*  (*f*[*i*]) to *f*′[*i*]. The DO executes the modification algorithm to perform the following modifications:

(1) finding the specific DCT that has the required block on the basis of the ranges of DCTs and then updating *V*_*i*_ = *V*_*i*_ + 1;
(2) generating a new block tag for modified data block by
  (10) Ti′=Sγ(f′[i]||(IDF||i||Li||Vi′)),Ci′=Sγ(IDF||i||Li||Vi′);
(3) sending the modification request message to the CSP, which includes (ID_*F*_, *i*, *f*′[*i*], *T*_*i*_′, *C*_*i*_′).

**Figure 2:**
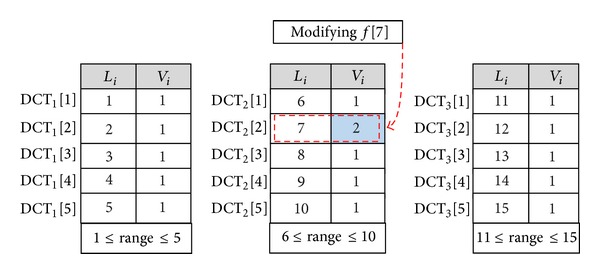
Performing modify operation on *f*[7] when the number of blocks in each table is 5.

Upon receiving the modification request message, the CSP replaces the block *f*[*i*] with *f*′[*i*] and updates the version of data block by replacing the tag (*T*_*i*_, *C*_*i*_) with (*T*_*i*_′, *C*_*i*_′).  Figure 2 shows that the data owner modifies block *f*[7] when the number of entities in each of table is 5.

*Data Insert.* To insert a new data block (f*[*i*]) after block (*f*[*i*]), the DO needs to run insert algorithm to perform the following modifications:

(1) finding the *i*th block of the file *F* by comparing its index with the range of DCTs;
(2) constructing a new row in the DCT after *i*th block and shifting the subsequent blocks (⌈*n*/*k*⌉ − *i*) one position down; the DO also sets the original index of data block *L*_*i*+1_* = *n* + 1 and the version number of the block *V*_*i*+1_* = 1 where *n* is number of blocks;
(3) the Do needs to increase maximum and minimum ranges of subsequent DCTs;
(4) generating a block tag (*T*_*i*+1_*, *C*_*i*+1_*) for the new data block by
  (11) Ti+1∗=Sγ(f∗[i+1]||(IDF||i+1||Li+1∗||Vi+1∗)),Ci+1∗=Sγ(IDF||i+1||Li+1∗||Vi+1∗);
(5) sending the insert request message to the CSP, which includes (ID_*F*_, *i* + 1, f*[*i*], *T*_*i*+1_*, *C*_*i*+1_*).

**Figure 3:**
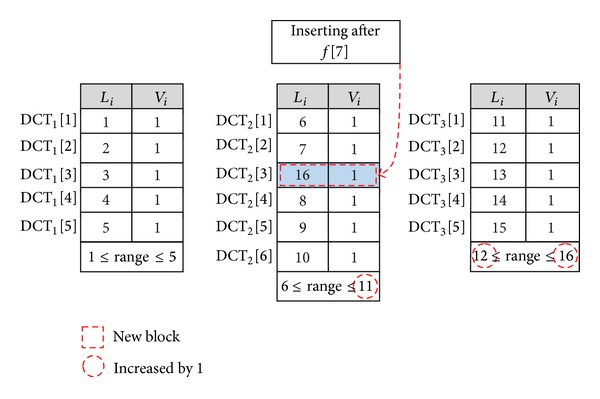
Inserting a new data block after *f*[7] when the number of blocks in each table is 5.

When the CSP receives such message, the new data block and the considering tag are inserted after position *i* in the file. For example, Figure 3 illustrates that the data owner only needs to shift 3 entities down to insert a new block (DCT_2_[3] = {16,1}) after block *f*[7] in the second table and increases all of range of next tables and the uprange of DCT_2_. 

**Figure 4:**
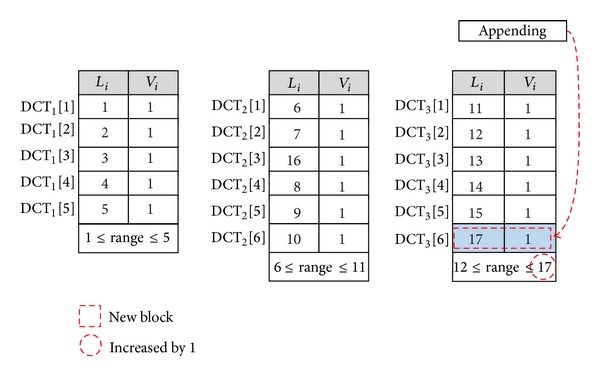
Appending a new data block.

*Data Append*. The append operation refers to the insertion of a new data block into the end of data blocks. Therefore, the Do only needs to insert a new row to the end of the last DCT without having to shift any entities of the DCTs. For instance, Figure 4 shows that to append a new block, the data owner only needs to create a free row for the last table and increase its uprange (UR_3_ = UR_3_ + 1). 

**Figure 5:**
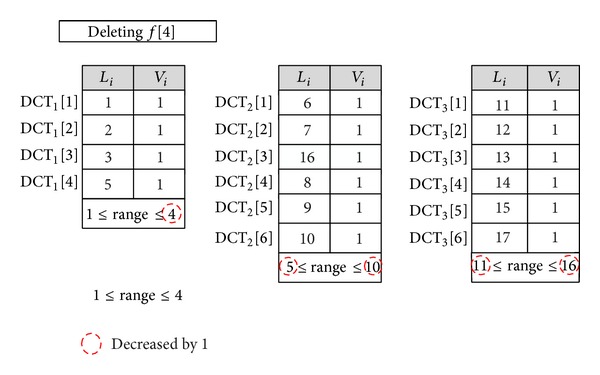
Deleting the *f*[4] when the number of blocks in each table is 5.

*Data Delete*. The delete operation is the opposite of the insert operation in which the *i*th block of the file of *F*  (*f*[*i*]) is removed. To achieve this goal, the DO finds the CDT that contains the required block on the basis of the DCT ranges. Then, the block is removed by shifting all of the subsequent blocks (⌈*n*/*k*⌉ − *i*) one position up. The DO sends a request to delete the *i*th block of the file of *F*. As it is shown in Figure 5, to delete a 4th data block (*f*[4]), the data owner only needs to shift up 1 row (*f*[5]) and the range of next tables will be reduced along with the uprange of the first table (UR_1_ = UR_1_ − 1).

## 5. Security Analysis

In this section, we evaluate the surety of our remote data auditing construction in term of security and correctness.

In the first step, we analyze the correctness of the verification algorithm. Upon receiving the challenge message ({*cs*_*i*_}_*i*=1_^*c*^), the CSP generates a pair (*μ*, *σ*) as a proof message. We extend (8) by using the properties of algebraic signature as follows:

(12) μ=∑i=cs1cscTi⊕Ci=∑i=cs1cscSγ(f[i]||(IDF||i||Li||Vi))⊕Sγ(IDF||i||Li||Vi)=∑i=cs1cscSγ(f[i])⊕rγSγ(IDF||i||Li||Vi)⊕Sγ(IDF||i||Li||Vi)=∑i=cs1cscSγ(f[i]).

When the DO obtains the proof message from the server, it verifies the proof message to ensure the storage correctness by using (9). We rewrite the equation on the basis of the algebraic signature properties to show why it is true:

(13) Sγ(σ)=Sγ(∑i=cs1cscf[i])=Sγ(f[cs1]+⋯+f[csc])=Sγ(f[cs1])+⋯+Sγ(f[csc])=∑i=cs1cscSγ(f[i])=μ.

Our scheme relies on the algebraic signature that generates a small entity as a signature for each block and is able to show any modifications in the original block. The algebraic signature also has the capability to verify a large amount of stored data on the distributed storage systems with minimum computation and communication overhead [35]. On the other hand, probability of collision in the algebraic signature is negligible [36]. For example, if the length of signature is 64 bits, the probability of collision is very small (2^−64^). As a result, the algebraic signature technique is useful for verifying the correctness of outsourced data specially by using the resource restricted devices.

## 6. Performance Analysis

In this section, we assess the performance of the proposed remote data auditing method. We also analyze the probability of misbehavior detection of this scheme. We give the computation complexity during the insert, delete, append, and modify operations and compare the results with the state-of-the-art remote data auditing methods proposed by Yang and Jia [17] and Wang et al. [19].

### 6.1. Probability of Misbehavior Detection

Our remote data auditing scheme is constructed on the basis of a random sampling strategy to reduce the workload on the server. In the sampling technique, the input file (*F*) is divided into several blocks (*n*) and a random number of blocks (*c*) are used to perform batch processing. We analyse the probability of misbehaviour detection of our scheme based on the block sampling.

Suppose the CSP modifies *m* blocks out of the *n* outsourced blocks. Then, the probability of corrupted blocks is equal to *p*_*m*_ = *m*/*n*. Let *c* be the number of blocks that the DO asks to verify the outsourced data in the challenge step and let *r* be the number of sectors in each block. Let *x* be a discrete random variable that indicates the number of blocks chosen by the DO that matches the blocks modified by the CSP. We compute the probability that at least one of the blocks picked by the DO matches one of the blocks modified by the server, namely, *P*_*x*_  (*x* ≥ 1), as follows:

(14) Px(x≥1)=1−Px(x=0)=1−(n−mn)·(n−m−1n−1)⋯(n−m−c+1n−c+1)=1−(1−nm)·(1−mn−1)⋯(1−mn−c+1)=1−∏i=0c−1(1−mn−i).

On one hand,

(15) (1−mn−i)≤(1−mn)⟹∏i=0c−1(1−mn−i)≤(1−mn)c⟹1−∏i=0c−1(1−mn−i)≥1−(1−mn)c.

Therefore,

(16) $$\begin{array}{c} \overset{\left(14),(15\right)}{⟹}P_{x}\left(x\geq 1\right)\geq 1-{\left(1-\frac{m}{n}\right)}^{c}=1-{\left(1-p_{m}\right)}^{c}. \end{array}$$

Since, each of the blocks consists of *r* sectors, such probability on the basis of sector corruption *p*_*s*_ is computed by

(17) pm≥1−(1−ps)r⟹(1−pm)c≤((1−ps)r)c⟹1−(1−pm)c≥1−(1−ps)rc⟹Px(x≥1)≥1−(1−ps)rc.

On the other hand,

(18) (1−mn−i)≥(1−mn−c+1)⟹∏i=0c−1(1−mn−i)≥(1−mn−c+1)c⟹1−∏i=0c−1(1−mn−i)≤1−(1−mn−c+1)c.

Therefore,

(19) $$\begin{array}{c} P_{x}\left(x\geq 1\right)\leq 1-{\left(1-\frac{m}{n-c+1}\right)}^{c}. \end{array}$$

Then, we can conclude that the probability of misbehavior detection is in

(20) $$\begin{array}{c} 1-{\left(1-\frac{m}{n}\right)}^{c}\leq P_{x}\left(x\geq 1\right)\leq 1-{\left(1-\frac{m}{n-c+1}\right)}^{c}. \end{array}$$

**Figure 6:**
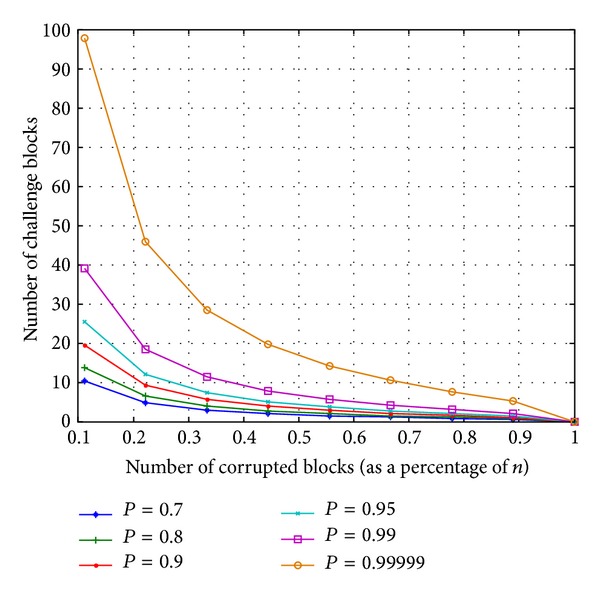
Number or required blocks as a challenge message under different number of data corruptions.

Suppose the DO divides 1 GB file into 125000 blocks 8 KB and outsources the blocks in the cloud.  Figure 6 shows the required number of challenge blocks (*c*) that are used to detect the different number of corrupted blocks (*m*) when the probability of misbehaviour detection is collected from a set of *P*_*x*_ = {0.7, 0.8, 0.9, 0.99, 0.99999}. For example, if the server modifies 0.1 of the outsourced blocks (*n*), the DO needs to randomly select 98 block as a challenge to achieve *P*_*x*_ of at least 0.99999. As it is clear, by increasing the number of corrupted blocks, the least number of challenge blocks is required to achieve such a probability of detection.

**Figure 7:**
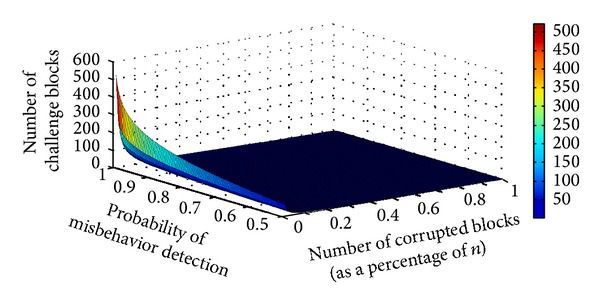
Number or required blocks as a challenge message under probability of misbehavior detection are from 0.5 to 1.

Figure 7 illustrates the number of challenge blocks when the probability of misbehavior detection is between 0.5 and 1 with variable rate of data corruption. For example, if the server modifies 0.01% out of the *n* outsourced blocks, the DO needs to randomly select 520 data blocks as a challenge for detecting the corrupted blocks with probability of 0.9899. It also can be seen that when the rate of corrupted blocks is more than 0.3%, the minimum numbers of challenge blocks are used to audit the outsourced data.

### 6.2. Evaluation and Experimental Results

Table 1 shows a comparison of our scheme and state-of-the-art remote data auditing protocols based on the communication and computation overhead through dynamic data update, where *n* is the number of blocks, *s* is the number of sectors of a block, *c* indicates the number of challenge blocks in each auditing query, and *k* indicates the number of the DCTs.

From the table, we can find that the Wang et al. method [19] has the maximum computation overhead during dynamic data update. In the Yang scheme [17], to insert a block after *i* or delete a specific block (*f*[*i*]), the verifier must shift (*n* − *i*) entities in the data structure. Therefore, the computation overhead of such method during insert and delete operations is *O*(*n*). We improve our auditing scheme by designing a new data structure (DCT) to reduce the computation overhead. As mentioned earlier in Section 4.2, the verifier only needs to shift (*n*/*k* − *i*) blocks that incurs *O*(*n*/*k*) computation overhead on the verifier. It is important to mention that to find a block (*f*[*i*]) in DCT structure, the verifier only needs to divide the location of block into *k* and find the appropriate DCT that incurs negligible overhead on verifier.

The first step to perform insert, delete, append, and modify operations is to identify that the ith data block of the file is going to be a part of which DCTs. The auditor is able to find the *i*th data block by computing the quotient of a division of the requested block index (*i*) by the number of data block in each DCT structure (*k*). Such quotient shows the DCT number and the remaining of the division shows the position of block in the found DCT. To insert a new data block after *j*th data block or delete the *j*th data block, the auditor has to find the considered DCT and the position of the block in it (*i*) and then moves forward or backward the remaining blocks of the DCT (*n*/*k* − *i*). Since each DCT contains (*n*/*k*) blocks, performing insert and delete operations incurs *O*(*n*/*k*) computation overhead on the auditor. The modification operation incurs *O*(*C*) as a computation overhead on the auditor. It is because the auditor only requires finding the position of *i*th data block in the DCTs and modifying the content. Finally, to append to an operation, the auditor must inset a new data block after the last data block of the last DCT which imposes *O*(*C*) as a computation cost.

We set up our own Eucalyptus private infrastructure as a service (IaaS) cloud in order to conduct this experiment using the existing IT infrastructure of center for mobile cloud computing (C4MCC). Eucalyptus is an acronym for “Elastic Utility Computing Architecture for Linking Your Programs to Useful Systems” and is actually a Linux-based open-source software architecture that can be installed without modification on all major Linux operating systems such as RHEL, Centos, Ubuntu, and Debian. The reason why we choose eucalyptus is due to its compatibility with Amazon AWS APIs [38] which means that we can use Eucalyptus commands to manage Amazon or Eucalyptus instances and move freely between an Eucalyptus private cloud and the Amazon Public cloud making it a hybrid cloud. Secondly, Eucalyptus cloud computing architecture is highly scalable because of its distributed nature and is flexible enough to support businesses of any size. Thirdly, it allows you to make your apps in-house on Eucalyptus and then migrate them to AWS; however, it was designed initially at the University of California, Santa Barbara, to support high performance computing (HPC) research [39]. The main components having their own Web-service interface that comprises our Eucalyptus installation are as follows.

1. Cloud controller (CLC) is actually the entry point into the cloud for administrators, managers, developers, and end-users and is accountable for satisfying the request of node managers. CLC is also responsible for making and implementing high level scheduling decisions with the help of cluster controllers.
2. Cluster controller (CC) generally executes on a computer system that has network connectivity to the systems running node controllers (NCs) and to the machine running the CLC. It actually manages a number of VMs and schedules their execution on particular NCs.
3. Node controller (NC) is executed on every system that is selected for hosting VM instances. It manages the life cycle of instances by making interaction with the OS and the hypervisor running on the same system and the CC.
4. Storage controller (SC) essentially implements block-accessed network storage such as EBS (Amazon Elastic Block Storage). Subsequently, it has the ability to send disk traffic across the local network to a remote storage site.
5. Walrus permits different users to store persistent data. It set access control policies for users to allow certain operations such as delete and create. Its interface is, however, compatible with Amazon's S3 to store and access both the virtual machine images and user data. It is actually a file-level storage system while essentially representing a block-level storage system.

We calculated the signature on the basis of defining multiplication in *GF*(2^*l*^) as polynomial multiplication modulo a generator polynomial. The multiplication by the unknown X is carried out by a left shifting and XORing with a parameter corresponding to the generator polynomial. As a result, a *γ* can be identified with the unknown so that multiplication by *γ* includes a left shift operation followed by a conditional XOR. Broder [40] proposed a technique to perform several shift operations in one time, by creating a table consisting of a number of decisions that are used as the XOR-operand. In this simulation, we assume that the length of a bit string (*l*) is 16 bits and the length of each block is 8 KB. We also divide each of the blocks into equal bit strings to compute the algebraic signature of each block.

**Figure 8:**
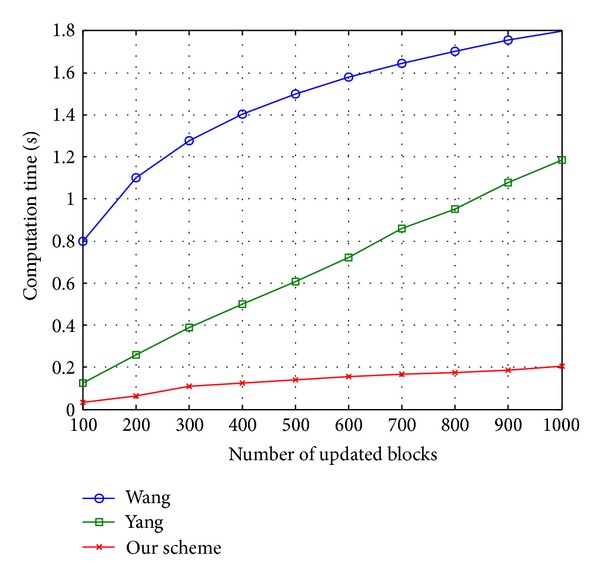
Comparison of computation cost under different number of update requests.

We conduct the experiments for updating an outsourced file (*F*) with length 1 GB, including 125,000 data blocks, and demonstrate the efficiency of the proposed scheme in Figure 8, where the numbers of updated (inserted or deleted) blocks are increasing from 100 to 1000 with intervals of 8. To insert or delete a block in the Wang scheme, the auditor needs to find the position of the block (*i*) in the MHT tree. Moreover, inserting or deleting a block needs to recalculate the hash of the root each time that incurs the huge computation overhead on the auditor. Similarly, in the Yang method, after finding the position of the block (*i*), as a precondition, the auditor has to shift the remaining (*n* − *i*) blocks for every insert or delete operations. Subsequently, repeating this process multiple (100–1000) times results in a significant computation overhead on the auditor. The proposed method considers 10 DCTs with size 12500 instead of a single array with size 125000 in the Yang scheme. Consequently, the number of shifts reduced in our method results in the minimum computation overhead on the client side. Figure 8 shows the performance in terms of computation cost under different number of update (insert or delete) operations. The analysis of the results shows the efficiency of our scheme.

**Figure 9:**
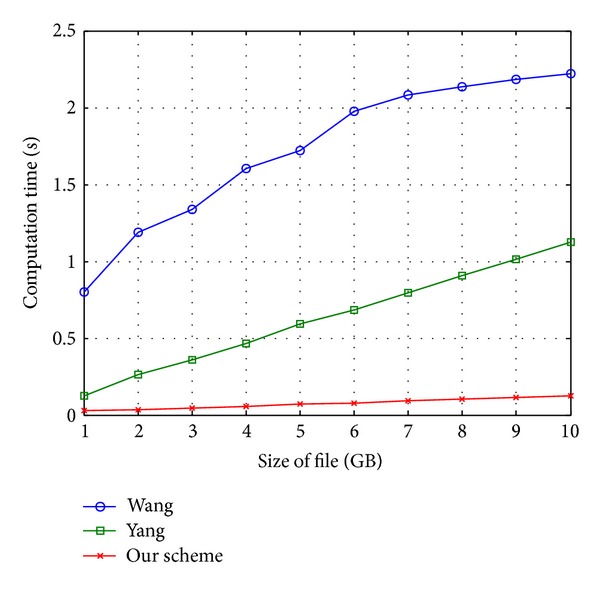
Comparison of computation cost under different file size from 1 GB to 10 GB when the number of update requests is 100.

We also show the impact of the size of the file on the computation overhead of the auditor by Figure 9, where the DO updates the different size of outsource data by inserting or deleting 100 blocks in random positions, respectively, from 1 to 10 GB file. The computation overhead of the Wang method is dramatically increasing from 0.8 to approximately 2.3 by increasing the size of file because the auditor encounters a huge number of data block in MHT. Similarly, in the Yang scheme, when the size of input file is enhancing from 1 GB to 10 GB with the same size of data block (8 kB), a number of data blocks are also increasing. Consequently, the auditor requires shifting a huge number of blocks to insert or delete a data block. As it is shown in Figure 9, our method incurs the minimum overhead on the auditor (maximum 0.2 sec when the size of file is 10 GB) due to using 10 DCTs instead of one while applying the algebraic signature. Therefore, our method can be applicable for auditing large scale files dynamically.

Figures 8 and 9 clearly show the performance and efficiency of our scheme in terms of computation overhead. The comparative analysis shows that our scheme is more efficient than Wang and Yang schemes, respectively.

## 7. Conclusion

In this paper, we present an efficient remote data auditing scheme to ensure the data storage security in cloud computing. To achieve this goal, we employed algebraic signature properties that empower our scheme to verify the integrity of outsourced data and reduce the computation overhead on the client and server side of the cloud. We also design a new data structure, namely, divide and conquer table, to support dynamic data update, including insert, delete, append, and modify operations. The divide and conquer table also allows the verifier to audit the large scale data and perform a large number of insert and delete operations with minimum computation overhead on the verifier and server. The security and performance analysis shows the efficiency and provability of our scheme.

As a part of future work, we extend our scheme to find the optimized number of divisions in the divide and conquer table. We also improve our scheme to be applicable for distributed cloud servers.

## Figures and Tables

**Table 1:** Comparison of different remote data auditing scheme.

Metric	Scheme		
	[19]	[17]	Our scheme
Communication	*O*(*c*log⁡*n*)	*O*(*c*)	*O*(*c*)
Computation Auditing			
Server	*O*(*c*log⁡*n*)	*O*(*cs*)	*O*(*cs*)
Verifier	*O*(*c*log⁡*n*)	*O*(*c*)	*O*(*c*)
Computation modify			
Verifier	*O*(*c*log⁡*n*)	*O*(*c*)	*O*(*c*)
Computation insert			
Verifier	*O*(*c*log⁡*n*)	*O*(*n*)	*O*(*n*/*k*)
Computation delete			
Verifier	*O*(*c*log⁡*n*)	*O*(*n*)	*O*(*n*/*k*)
Computation append			
Verifier	*O*(*c*log⁡*n*)	*O*(*c*)	*O*(*c*)
